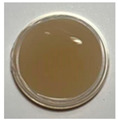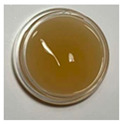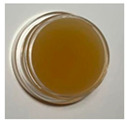# Hydrogel Containing *Borassus flabellifer* L. Male Flower Extract for Antioxidant, Antimicrobial, and Anti-Inflammatory Activity

**DOI:** 10.3390/gels8020126

**Published:** 2022-02-17

**Authors:** Prakairat Tunit, Phanit Thammarat, Siriporn Okonogi, Chuda Chittasupho

**Affiliations:** 1 Thai Traditional Medicine Program, Faculty of Nursing and Allied Health Sciences, Phetchaburi Rajabhat University, Phetchaburi 76000, Thailand; prakairat.tun@mail.pbru.ac.th; 2 Department of Pharmaceutical Sciences, Faculty of Pharmacy, Chiang Mai University, Chiang Mai 50200, Thailand; phanit.thamma@cmu.ac.th (P.T.); siriporn.okonogi@cmu.ac.th (S.O.); 3 Research Center of Pharmaceutical Nanotechnology, Faculty of Pharmacy, Chiang Mai University, Chiang Mai 50200, Thailand

**Keywords:** *Borassus flabellifer* L., antioxidant activity, antibacterial activity, *Cutibacterium acnes*, gel

## Abstract

*Borassus flabellifer* L. is a plant in Arecaceae family, widely distributed and cultivated in tropical Asian countries. The purpose of this study was to identify the bioactive compounds of *B.* *flabellifer* L. male flower ethanolic extract and investigate the antioxidant, anti-inflammatory, and antibacterial activities against *Cutibacterium acnes*. Total phenolic compounds and total flavonoids in *B.* *flabellifer* L. male flower ethanolic extract were determined by the Folin–Ciocalteu method and aluminum chloride colorimetric assay, respectively. Active substances in the extract and their quantities were analyzed by liquid chromatography and mass spectrometry (LC–MS/MS). The antioxidant evaluation was carried out using DPPH, ABTS free radical scavenging assays, and FRAP assay. *C. acnes* inhibitory activity was performed by the broth microdilution method. Anti-inflammatory activity was determined by the protein denaturation assay. In addition, gel containing different amounts of *B.* *flabellifer* L. male flower extract was formulated. The physical stability of the gel was observed by measuring viscosity and pH after six heating and cooling cycles, as well as 1-month storage at 4, 30, and 45 °C. The total phenolic content in the extract was 268.30 ± 12.84 mg gallic acid equivalent/g crude dry extract. The total flavonoid contents in the extract were 1886.38 ± 55.86 mg quercetin equivalent/g extract and 2884.88 ± 128.98 mg EGCG equivalent/g extract, respectively. The LC–MS/MS analysis revealed the presence of gallic acid, coumarin, and quercetin and the concentrations of quercetin, coumarin, and gallic acid in *B. flabellifer* male flower ethanolic extract were 0.912, 0.021, and 1.610 µg/mL, respectively. DPPH and ABTS antioxidant assays indicated that the *B.* *flabellifer* L. male flower extract had IC_50_ values of 31.54 ± 0.43 and 164.5 ± 14.3 µg/mL, respectively. FRAP assay revealed that the *B.* *flabellifer* male flower extract had high ferric ion reducing power. The extract was able to inhibit *C.* *acnes* bacteria with a minimum inhibitory concentration (MIC) of 250 mg/mL. At 250 and 500 µg/mL, the extract demonstrated the highest anti-inflammatory activity. The gel containing 31.25% *w/w* and 62.5% *w/w* showed good physical stability after six heating and cooling cycles, as well as 1-month storage.

## 1. Introduction

Acne vulgaris is a common skin disorder in which the skin’s pores are blocked by sebum, bacteria, and dead cells. Acne is associated with inflammation of the pilosebaceous unit. Previous studies reported that oxidative stress components, such as reactive oxygen species and lipid peroxide are involved in the pathogenesis and progression of the disease [1]. In addition, acne vulgaris is triggered by *Cutibacterium acnes* (*C.* *acnes*) under the influence of normal circulating dehydroepiandrosterone. *C.* *acnes* is a Gram-positive bacteria that lives in the sebaceous follicle. It is one of the main causes of acne vulgaris by inducing inflammation and follicular epidermal proliferation [2]. Inhibition of *C.* *acnes* by topical antibacterial medications reduces its entry into the skin and prevents acne from spreading to other areas. 

The natural antioxidant systems in the skin, including superoxide dismutase and catalase enzymes, maintain the balance of cellular redox reactions. Elevated reactive oxygen species (ROS) levels and decreased antioxidant levels can lead to oxidative stress and cause cellular membrane. Al-Shobaili showed a significant elevation of plasma lipid peroxide levels in acne patients compared with healthy volunteers [3]. In addition, the activities of superoxide dismutase and catalase enzymes and the level of total antioxidant capacity significantly diminished in acne patients [3].

Various acne vulgaris treatments target different steps in the pathogenesis of acne, including reducing *C.* *acnes* proliferation, suppressing androgens, and decreasing sebum production to prevent follicular occlusion [4]. The treatment of acne using topical antibacterial agents, such as clindamycin and erythromycin, is effective and faster than hormonal adjustments and laser treatments. However, the long-term use of topical antibiotics to treat acne vulgaris can cause undesirable side effects and induce *C.* *acnes* resistance. *C.* *acnes* resistance to antibiotics, such as erythromycin and clindamycin has been detected with high prevalence in Mediterranean countries mainly due to antibiotic abuse [5,6,7,8]. Side effects of topical antibiotic use are dryness of the treated area, skin irritation, and contact dermatitis, including red, dry, and itchy skin. Many plants have shown antibacterial and anti-inflammatory activities. Natural active compounds are attractive for use as a combination or replacement of antibacterial drugs to treat acne vulgaris since they possess fewer side effects and have multiple mechanisms of action [9].

*Borassus flabellifer* L. (*B.* *flabellifer*) (Arecaceae) is a plant widely grown in Southeast Asia [10]. Folk medicine uses various parts of *B.* *flabellifer* as a diuretic, antimicrobial, tonic, laxative, and wound healing agent [11]. A study on the pharmacological activity of the male flower found that the ethanol extracts at concentrations of 150 and 300 mg/kg had anti-inflammatory [12], analgesic, and antipyretic effects in rats [13]. The root and male flower parts extracted with methanol were found to have antioxidant activity [14]. However, the bioactive compounds, antioxidant, and anti-*C.* *acnes* activities of male flower ethanolic extract have never been reported. The objective of this study was to quantify phenolic compounds and flavonoids, as well as the active substances in the ethanol extract from the male flower of *B.* *flabellifer.* The antioxidant, anti-*C.* *acne*, and anti-inflammatory potential were evaluated. Gel containing different concentrations of *B.* *flabellifer* extract was developed and investigated for its physical stability.

## 2. Results and Discussion

### 2.1. Yield of B. flabellifer Male Flower Ethanolic Extract and Total Phenolic Content in B. flabellifer Male Flower Ethanolic Extract

**Figure 1:**
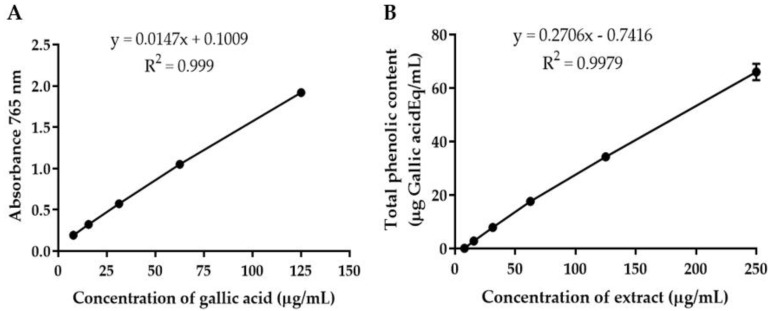
(**A**) Calibration curve of gallic acid. (**B**) Total phenolic content of *B. flabellifer* male flower ethanolic extract.

The yield of ethanolic extract obtained from dried *B.* *flabellifer* male flower was measured. The yield was reported as 5.06 ± 1.35% *w/w*. The total phenolic content in *B.* *flabellifer* male flower ethanolic extract using the Folin–Ciocalteu reagent was expressed in terms of gallic acid equivalent. The standard curve equation was y = 0.0147x + 0.1009, r^2^ = 0.9990 (Figure 1A). The results showed that total phenolic content increased with the concentration of *B.* *flabellifer* male flower extract with a correlation coefficient of 0.9979 (Figure 1B). The average total phenolic content in the extract was 268.30 ± 12.84 mg gallic acid equivalent/g crude dry extract, calculated from 31.25–250 µg/mL crude extract. The total phenolic content in plant extracts of *B.* *flabellifer* male flower ethanolic extract depends on the polarity of solvent used for extraction [15]. Tusskorn et al. reported that *B.* *flabellifer* flowers extracted with methanol and ethyl acetate had phenolic contents of 159.3 ± 0.3 and 90.0 ± 0.0 mg GAE/g extract, respectively [16]. The results suggested that the total phenolic content in *B.* *flabellifer* male flower’s ethanolic extract was significantly greater than the extracts from methanol and ethyl acetate. The total phenolic content of *B.* *flabellifer* root extract from ethanol was significantly higher than the petroleum ether extract [17]. These results agreed with our results and previous reports, showing that the total phenolic compound in terms of the gallic acid equivalent amount was significantly higher when *B.* *flabellifer* was extracted with ethanol.

### 2.2. Total Flavonoid Content in B. flabellifer Male Flower Ethanolic Extract

**Figure 2:**
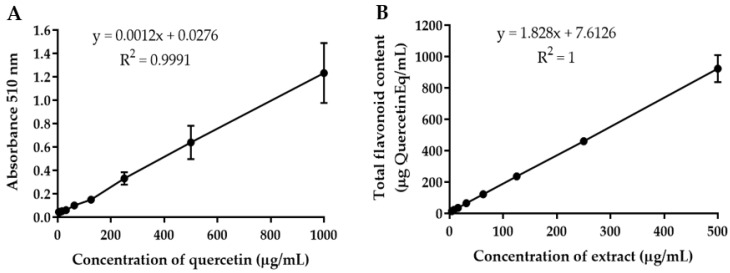
(**A**) Calibration curve of quercetin. (**B**) Total flavonoid content of *B.* *flabellifer* male flower ethanolic extract.

**Figure 3:**
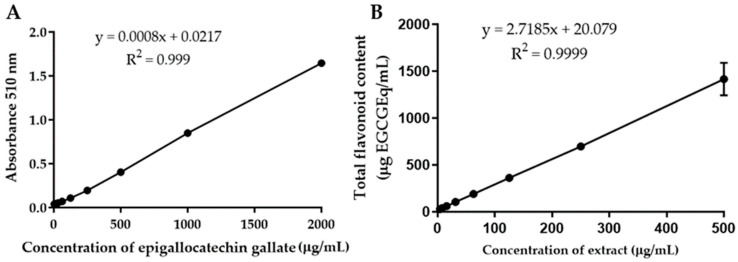
(**A**) Calibration curve of epigallocatechin gallate. (**B**) Total flavonoid content of *B.* *flabellifer* male flower ethanolic extract.

The total flavonoid content in *B.* *flabellifer* male flower ethanolic extract determined by the aluminum chloride colorimetric method was expressed in quercetin and epigallocatechin (EGCG) equivalent amounts. The quercetin standard curve equation was y = 0.0012x + 0.0276, r^2^ = 0.9991 (Figure 2A). The EGCG standard curve equation was y = 0.0008x + 0.0217, r^2^ = 0.999 (Figure 3A). The concentration of total flavonoids in the extract increased with the concentration of the extract (Figure 2B and Figure 3B). The average total flavonoid contents in the extract were 1886.38 ± 55.86 mg quercetin equivalent/g extract and 2884.88 ± 128.98 mg EGCG equivalent/g extract, calculated from 62.5–500 µg/mL of crude extract. These results indicated that the ethanolic extract of male flowers contained a high amount of flavonoids. The total flavonoid contents found in each part of *B.* *flabellifer* were different. The lower polar solvent could extract higher total flavonoid content from *B.* *flabellifer.* The root and fruit extracts from ethanol yielded higher total flavonoid concentrations at 3.57 ± 1.26 and 7.02 ± 0.61 mg quercetin equivalent/g of crude extract, respectively. In contrast, the root extract from petroleum ether contained 17.41 ± 1.89 mg quercetin equivalent/g of crude extract. In addition, part of *B.* *flabellifer* contained different amounts of total flavonoids. The total flavonoid content in quercetin equivalent in *B.* *flabellifer* flower was significantly higher than the root [18]. 

### 2.3. Analysis of B. flabellifer Male Flower Ethanolic Extract Phytochemical Component by LC–MS/MS

**Figure 4:**
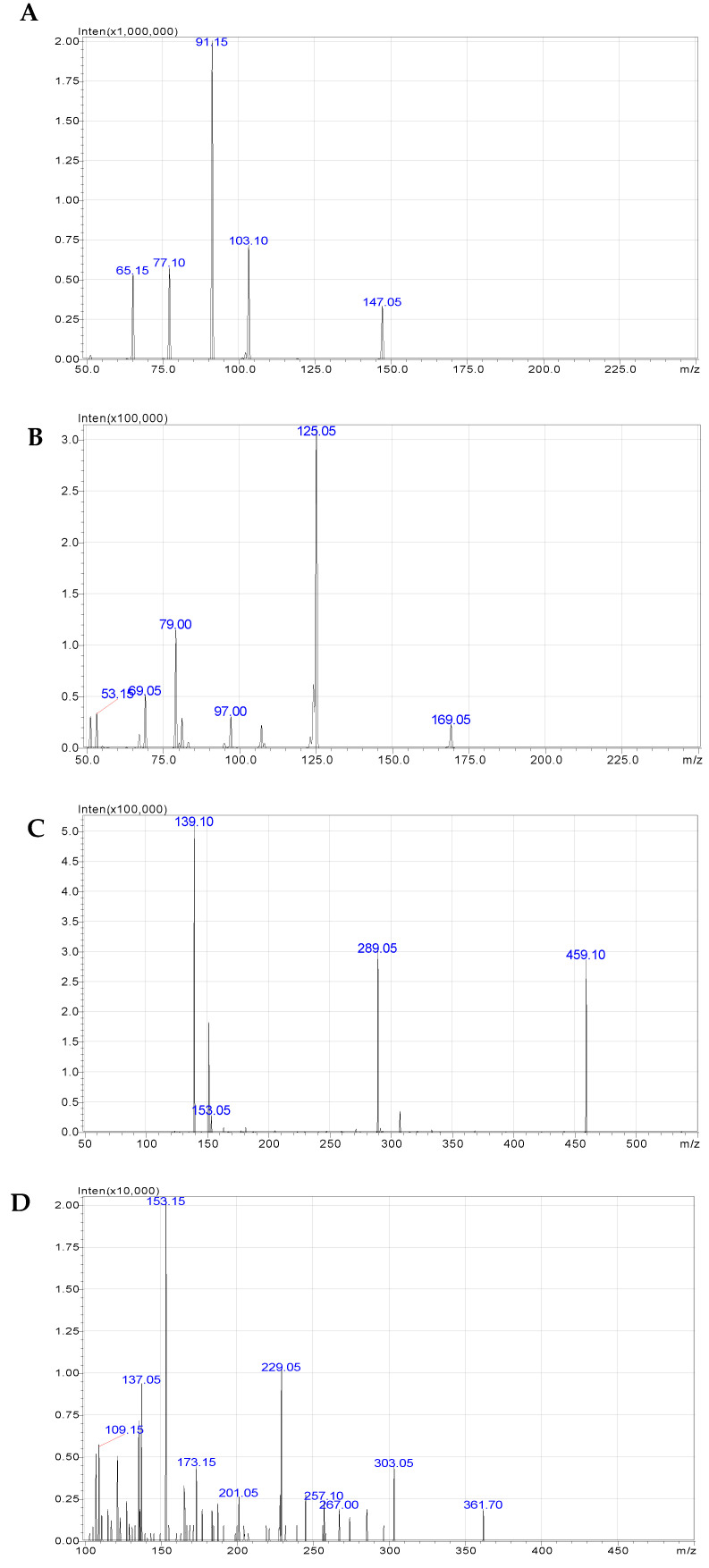
LC–MS/MS analysis presenting the precursor ion and product ion spectra of (**A**) coumarin, (**B**) gallic acid, (**C**) EGCG, and (**D**) quercetin.

**Figure 5:**
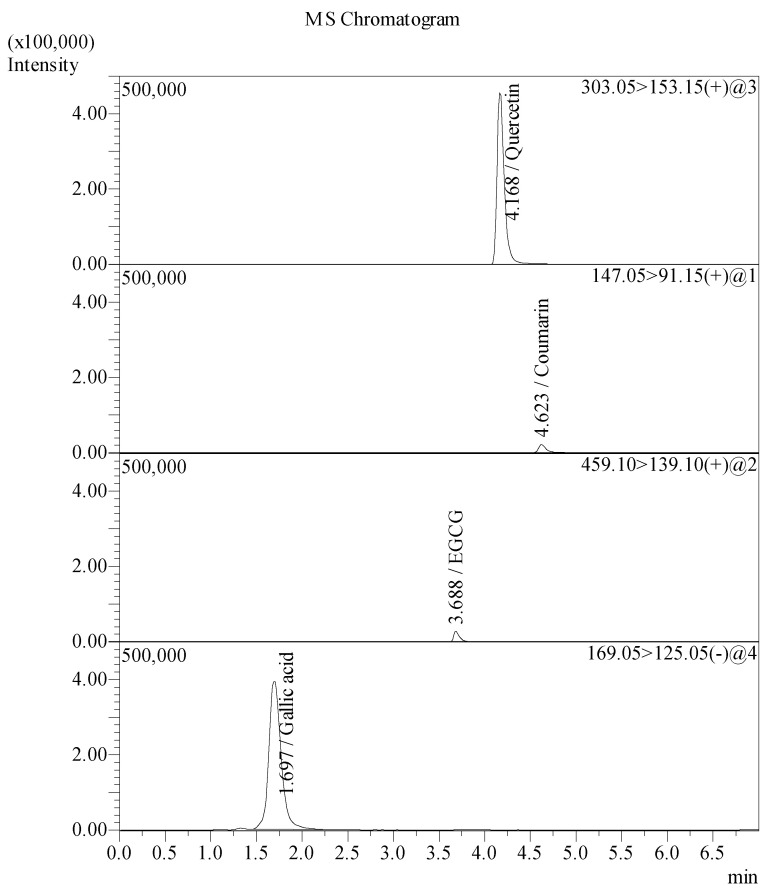
Chromatograms of standard compounds determined by LC–MS/MS.

A total of four bioactive compounds in *B. flabellifer* male flower ethanolic extract were identified and characterized by a correlation of the molecular (precursor) ions and the fragmentation patterns (product ions) acquired by LC–MS/MS analysis (Figure 4). The LC–MS/MS data were compared with the molecular ions and fragmentation patterns of the reference standards. The results showed that the bioactive compounds identified in *B. flabellifer* male flower ethanolic extract were quercetin, coumarin, and gallic acid, as shown in Figure 5.

**Figure 6:**
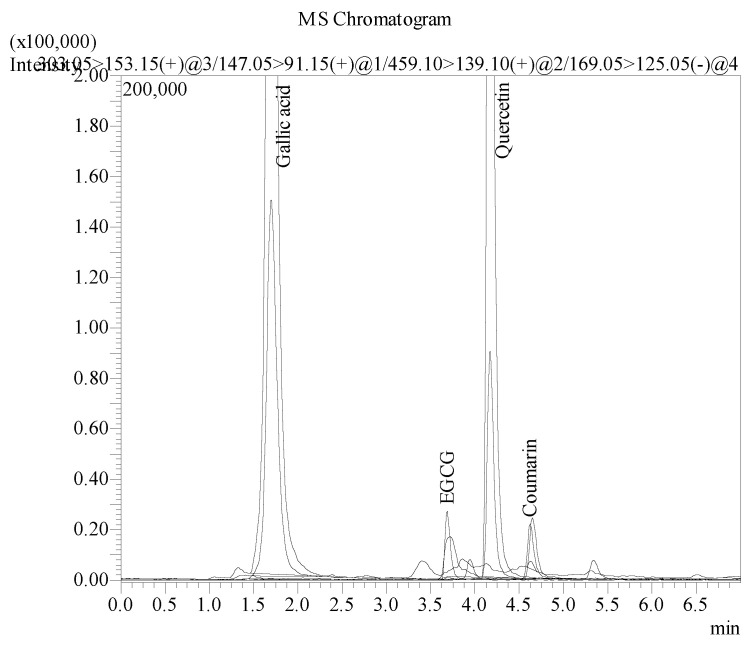
Overlay of LC–MS/MS extracted ion chromatograms (EIC) for *B.* *flabellifer* male flower ethanolic extract.

The main peaks of quercetin, coumarin, and EGCG reference standards were revealed at retention times of 4.173, 4.636, and 3.686 min corresponding to ion transitions of the precursor to the protonated ions [M-H]+ at *m/z* 303.15 → 153.15, 147.05 → 91.15, and 459.10 → 139.10, respectively. The main peak of gallic acid reference standard was shown at a retention time of 1.700 min. The ion transition of the precursor to the product ion was deprotonated ions [M-H] at *m/z* 169.05 → 125.05. The results of the LC–MS/MS analysis of *B. flabellifer* male flower ethanolic extract are presented in Figure 6 and Table 1. The LC–MS/MS analysis confirmed the presence of quercetin, coumarin, and gallic acid in *B. flabellifer* male flower ethanolic extract. In addition, the concentrations of quercetin, coumarin, and gallic acid in *B. flabellifer* male flower ethanolic extract were 0.912, 0.021, and 1.610 µg/mL, respectively.

### 2.4. The 2,2-Diphenyl-1-picrylhydrazyl (DPPH) Free Radical Scavenging Activity of B. flabellifer Male Flower Ethanolic Extract

**Figure 7:**
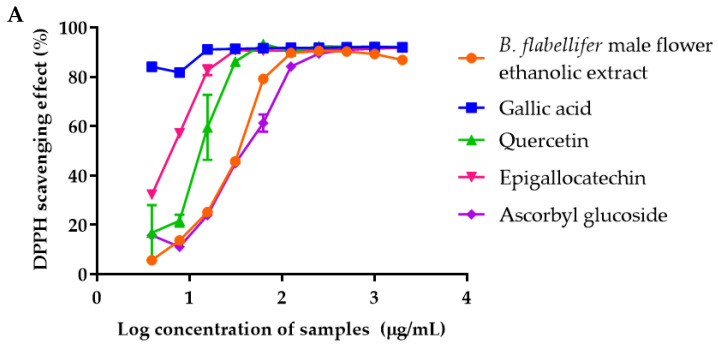

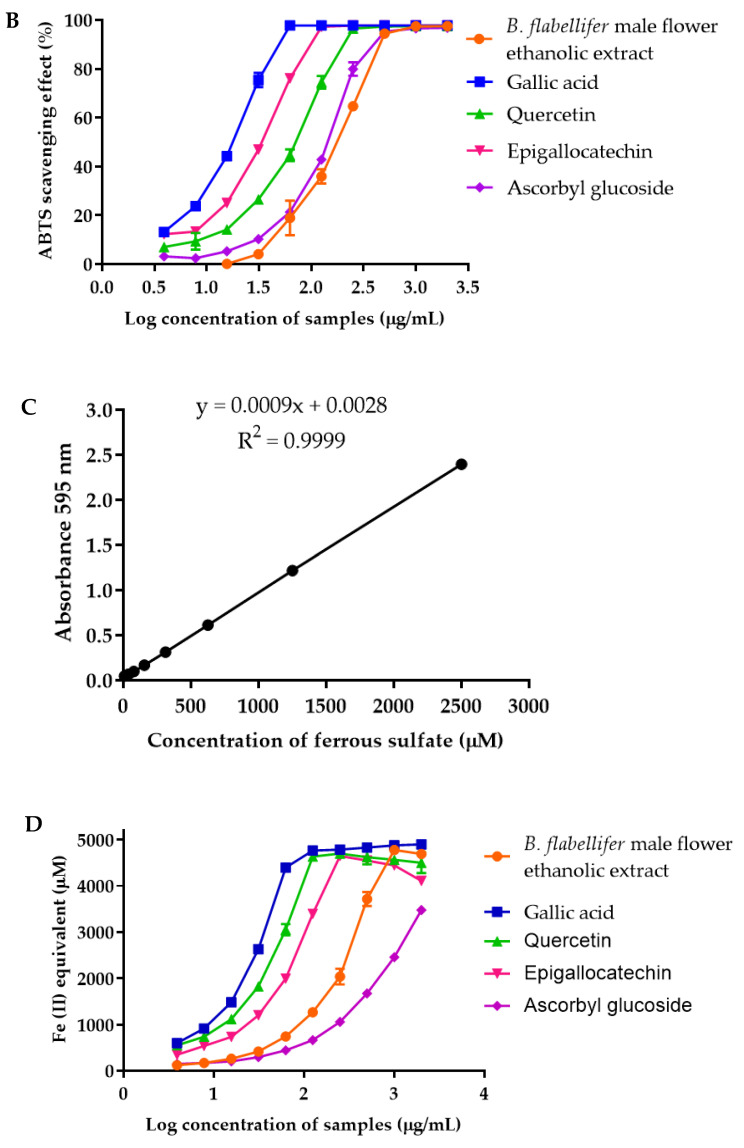
The antioxidant activity of gallic acid, quercetin, epigallocatechin, ascorbyl glucoside, and *B.* *flabellifer* flower ethanolic extract determined by (**A**) DPPH free radical scavenging assay, (**B**) ABTS free radical scavenging assay, (**C**) standard curve of ferric reducing antioxidant power assay using ferrous sulfate, (**D**) FRAP assay.

The *B.* *flabellifer* male flower extract showed DPPH free radical scavenging activity with the IC_50_ value of 31.54 ± 0.43 µg/mL. The IC_50_ values of gallic acid, quercetin, EGCG, and ascorbyl glucoside were <3.9, 13.77 ± 2.80, 6.25 ± 0.19, and 38.68 ± 2.00 µg/mL, respectively. These results indicated that the potency of antioxidants to scavenge the DPPH radical was in the following order: Gallic acid > EGCG > quercetin > *B.* *flabellifer* male flower extract > ascorbyl glucoside (Figure 7A). The DPPH scavenging activities of *B.* *flabellifer* male flower extracted by different types of solvents have been reported. Tusskorn et al. showed that the male flower of *B.* *flabellifer* extracted by ethyl acetate and chloroform had the IC_50_ values of 287.03 ± 6.90 and >1000 µg/mL, respectively [16]. Kavatagimath et al. reported that the *B.* *flabellifer* male flower extracted by 95% ethanol and butylated hydroxytoluene had the IC_50_ values of 460 and 419 µg/mL, respectively [18]. The IC_50_ values of *B.* *flabellifer* male flower ethanolic extract reported in our study showed a significantly lower value indicating that the male flower ethanolic extract had a superior antioxidant activity compared with the other solvents.

The superior antioxidant activity of *B. flabellifer* male flower extract obtained from our study might be due to the lower degradation extent of phytochemical compounds, i.e., polyphenolic compounds (phenolic compounds and flavonoids) in the extract during the extraction process. The polyphenolic compounds can degrade upon drying, extraction, and long-term storage [19]. According to the Kavatagimath et al. report, the extraction temperature might affect the stability of phenolic compounds, which are the active pharmaceutical ingredient for antioxidant activity [18]. The high temperature during continuous hot extraction using Soxhlet apparatus may result in degradation of the phenolic compounds and reduced antioxidant activity observed by the DPPH method [20]. Kotsiou et al. discussed that the main reason for phenolic compound degradation under high temperature was oxidation and hydrolysis reactions [21]. In addition, the age of *B. flabellifer* flower and the storage time of flower extract had been shown to influence the amount and stability of phenolic compounds in the extract, respectively [20,22]. Therefore, using mature (6 month-old) *B. flabellifer* flower, cold maceration, and freshly prepared extract as an active ingredient in the formulation was recommended to achieve the best antioxidant activity.

### 2.5. The 2,2′-Azino-bis(3-ethylbenzothiazoline-6-sulfonic acid) (ABTS) Free Radical Scavenging Activity of B. flabellifer Male Flower Ethanolic Extract

The *B.* *flabellifer* male flower ethanolic extract showed the scavenging activity of the ABTS^+^ radical. The IC_50_ value of *B.* *flabellifer* male flower extract for ABTS free radical scavenging activity was 164.5 ± 14.3 µg/mL. The IC_50_ values of gallic acid, quercetin, EGCG, and ascorbyl glucoside were 16.11 ± 0.33, 61.92 ± 3.01, 27.21 ± 6.00, and 131.20 ± 3.91 µg/mL, respectively. The results indicated that the potency of antioxidants to scavenge ABTS radical was in the following order: Gallic acid > EGCG > quercetin > ascorbyl glucoside > *B.* *flabellifer* male flower extract (Figure 7B). The IC_50_ values of *B.* *flabellifer* male flower methanolic and ethyl acetate extract, determined by the ABTS free radical scavenging assay, were 10.82 ± 0.78 and 462.92 ± 25.70 µg/mL, respectively [16]. These results suggested that the *B.* *flabellifer* male flower extracted in this study yielded higher antioxidant activity compared with the previous report.

Do et al. reported the effect of plant extraction solvent on total phenolic content, total flavonoids content, and antioxidant activity [23]. They concluded that the best extracting solvent to yield the highest total phenolic compound and total flavonoids content in the extract was ethanol. The different solvents used in extraction resulted in the differences in compositions and antioxidant activities of the extracts. Ethanol (95%) is suitable for extracting bioactive compounds with a broader range of polarity, when compared with methanol (more polarity) and ethyl acetate (less polarity). Since the Pearson correlation showed that both phenolic and flavonoid contents were directly proportional to their antioxidant activity, the decrease in polyphenolic compounds resulted in the decrease in antioxidant activity. Therefore, these results imply that 95% of ethanol may be the most appropriate solvent to extract polyphenolic compounds from *B. flabellifer* male flower.

### 2.6. Ferric Reducing Antioxidant Potential (FRAP) of B. flabellifer Male Flower Ethanolic Extract

The FRAP assay was performed to determine the reducing capacity of *B.* *flabellifer* male flower extract in a redox reaction. The results revealed good linearity of ferrous sulfate obtained within the range of 9.8–2500 µM (r^2^ = 0.9999) (Figure 7C). The results of FRAP assay were expressed as Fe^2^^+^ equivalent. Gallic acid, quercetin, EGCG, ascorbyl glucoside, and *B.* *flabellifer* male flower extract at 1 mg/mL exhibited 4877.13 ± 34.43, 4566.46 ± 59.81, 4442.79 ± 42.72, 2458.42 ± 41.93, and 4778.25 ± 56.20 µM Fe^2^^+^ equivalent, respectively (Figure 7D). The results indicated that the potency of antioxidants to reduce ferric ion was in the following order: Gallic acid > *B.* *flabellifer* male flower extract > quercetin > EGCG > ascorbyl glucoside. These results indicated that compared with the other mechanisms, the antioxidant activity of *B.* *flabellifer* male flower extract was mainly based on the reducing power of the compounds in the extract, which reduced ferric ion (Fe^3^^+^) to the ferrous ion (Fe^2+^).

### 2.7. Pearson Correlation of Total Phenolic and Flavonoid Contents with Antioxidant Activities of B. flabellifer Male Flower Ethanolic Extract

Pearson correlation coefficients for total phenolic and flavonoid contents with *B.* *flabellifer* male flower ethanolic extract antioxidant activities were shown in Table 2. Total phenolic and flavonoid contents positively and significantly correlated with the antioxidant activities measured by ABTS and FRAP assays. In addition, the results showed a correlation between the total phenolic content and antioxidant activity determined by the DPPH scavenging assay. There was no correlation between the total flavonoid content and DPPH free radical scavenging activity for *B.* *flabellifer* male flower ethanolic extract. These results indicated that the total phenolic and flavonoid contents contributed to the antioxidant activities of *B.* *flabellifer* male flower ethanolic extract.

The polyphenols and flavonoids present in the extract from the male flower of *B.* *flabellifer* exert several antioxidant properties through several mechanisms, including free radical scavenging, reduction potential, and metal chelation. The antioxidant activity of polyphenols was associated with the capability to inactivate reactive radical species due to the neutralization of free radicals. Neutralization occurs when polyphenols transfer their electrons and/or hydrogen atoms to radicals. The reduction potential of polyphenols resulted from the reduction of Fe^3^^+^ to Fe^2^^+^. The reduction potential of polyphenols depends on the presence of double bonds in the rings and the number of OH groups. In addition, chelation of Fe^3^^+^ may be another possible antioxidant mechanism of polyphenolic compounds containing two or more groups of OH groups [24].

The antioxidant capacity of a plant crude extract could be a combined effect of phenolic, flavonoids, and other reducing compounds, including gallic acid, coumarin, and quercetin. In the present study, the antioxidant activity was measured by DPPH, ABTS radical scavenging assay, and FRAP assay. The extract from the male flower of *B.* *flabellifer* showed a significantly higher radical scavenging activity and ferric chloride reducing power compared with the previous reports. In addition, the radical scavenging activity of the extract was in accordance with the phenolic and flavonoid contents in the extract. The higher value of total phenolic and flavonoid contents might be responsible for the potent antioxidant activity of this extract. This might be due to the drying process and extraction method of the plant that preserve the bioactive compounds.

The selective HPLC–DPPH post-column methodology might be another interesting option to investigate the antioxidant activity of the bioactive compounds containing *B. flabellifer* L. male flower extract [25].

### 2.8. Anti-Cutibacterium Acnes Activity of B. flabellifer Male Flower Ethanolic Extract

The minimum inhibitory concentration (MIC) of *B.* *flabellifer* male flower ethanolic extract against *C.* *acnes* was 250 mg/mL. In comparison, the standard antibacterial drug, i.e., clindamycin, had the minimum inhibitory concentration (MIC) of *C.* *acnes* as 0.781 µg/mL. The results suggested the use of *B.* *flabellifer* male flower ethanolic extract to treat acne vulgaris caused by *C.* *acnes* infection. Wongmanit et al. reported the antibacterial activity of *B.* *flabellifer* male flower ethanolic extract against *Enterococcus faecalis* and *Staphylococcus aureus*, but did not report the activity against *Escherichia coli* [22]. Alamelumangai et al. reported that the methanolic and ethanolic seed coat of *B. flabellifer* extract at 50 mg/mL could inhibit the growth of *Aspergillus brasiliensis* and *Bacillus subtilis* [26]. Reshma et al. isolated 2,3,4-trihydroxy-5-methylacetophenone from palm juice extract, which showed a broad-spectrum antibacterial activity against *Escherichia coli, Mycobacterium smegmatis, Staphylococcus aureus,* and *Staphylococcus simulans* [27].

Some phenolic compounds in *B. flabellifer* L. male flower extract, including gallic acid, quercetin, and coumarin have shown antibacterial activity against *C. acnes* [28]. Gallic acid is a hydroxybenzoic acid that inhibited bacterial growth by inducing irreversible changes in membrane properties of both Gram-positive and Gram-negative bacteria [29]. Gallic acid has shown a strong anti-*C. acnes* activity by the inhibition of lipase enzyme [30]. Coumarin and quercetin showed a significant antibacterial effect against *C. acnes* [31]. The mechanism of antibacterial activity of coumarin was inhibiting the bacterial fatty acid synthesis pathway [32]. The antibacterial mechanism of quercetin included destroying bacterial cell wall, changing cell permeability, affecting protein synthesis and expression, reducing enzyme activities, and inhibiting nucleic acid synthesis [33].

### 2.9. Effect of B. flabellifer Male Flower Ethanolic Extract on Inhibition of Protein Denaturation

The anti-inflammatory activity of *B. flabellifer* male flower ethanolic extract was evaluated against denaturation of egg albumin. The *B. flabellifer* male flower ethanolic extract at 250, 500, and 1000 µg/mL inhibited albumin denaturation of 47.55 ± 1.30, 47.79 ± 3.53, and 27.40 ± 5.08%, respectively. Diclofenac diethylammonium, a positive control, inhibited albumin denaturation to a greater extent than the extract with the IC_50_ of 0.26 ± 0.02 mg/mL. Denaturation of protein results in autoantigen generation, leading to inflammation. Inhibition of protein denaturation might inhibit an inflammatory activity. The results suggested that the *B. flabellifer* male flower ethanolic extract at 500 µg/mL was the optimal concentration for anti-inflammatory activity. The inhibition of *B. flabellifer* male flower ethanolic extract and diclofenac diethylammonium at higher than 500 and 2000 µg/mL, respectively, showed a decreasing inhibitory activity. These results agreed with the previous report showing a decreasing inhibition rate of ibuprofen [34].

Protein denaturation may cause auto-antigens, leading to inflammatory responses. The substances that can protect protein from denaturation may be used for developing an anti-inflammatory agent. The inhibition of albumin denaturation assay has been widely used for determining an in vitro anti-inflammatory assay [35,36,37,38]. *B.* *flabellifer* male flower ethanolic extract at 250 and 500 µg/mL was shown to inhibit albumin protein denaturation. The inhibition decreased with the increasing concentration of *B.* *flabellifer* male flower ethanolic extract. The mechanism of protein denaturation inhibition of the flower extract might be due to the interaction of polyphenolic compounds in the extract and albumin protein, resulting in improved thermal stability of proteins. Binding of gallic acid with protein increased protein intramolecular packing and induced higher thermal stability [39]. Coumarin and its derivatives exhibited an inhibition of heat-induced protein denaturation at specific concentrations [40,41]. The anionic form of quercetin was reported to bind a folded protein and increased the thermal stability of the planar conformation [42]. The interaction of polyphenols and protein may result in secondary structural changes in protein due to the reduction of surface hydrophobicity. Therefore, increasing the extract concentration containing an higher amount of polyphenols might result in a negative effect on the structural and thermal properties of protein [43].

### 2.10. Evaluation of Physical Characteristics and Stability of Gel Containing B. flabellifer Male Flower Ethanolic Extract

The freshly prepared gel containing *B. flabellifer* male flower ethanolic extract was characterized as a light brown gel with a smooth texture and the distinctive odor of *B. flabellifer* male flower ethanolic extract. The gel had a darker color when the concentration of the flower extract increased (Table 3). The gel containing 25, 31.25, and 62.5% *w/w* of *B. flabellifer* male flower ethanolic extract had pH values of 6.05 ± 0.01, 6.31 ± 0.02, and 6.59 ± 0.02, respectively.

**Figure 8:**
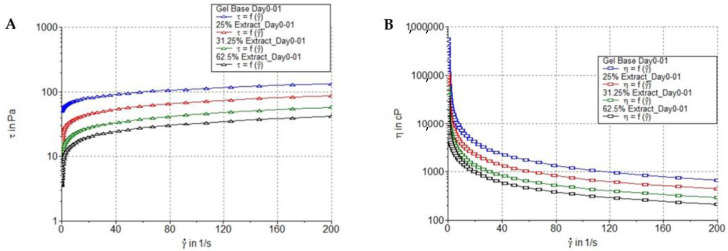
Rheology measurement. (**A**) Flow curves of gel base, gel containing 25, 31.25, and 62.5% *w/w* extract expressed as shear rate and applied shear stress. (**B**) Flow curves of gel base, gel containing 25, 31.25, and 62.5% *w/w* extract expressed as viscosity and shear rate.

A plot of viscosity versus shear rate for the gel base and containing *B. flabellifer* male flower ethanolic extract is shown in Figure 8. The results clearly indicated that the apparent viscosity of the gel base and gel containing *B. flabellifer* male flower ethanolic extract decreased significantly with the increasing shear rate, i.e., from around 100,000 cP at a shear rate of 0.9 s^−1^ to below 1000 cP at a shear rate of 200 s^−1^, indicating that the gel exhibited a pseudoplastic character. The viscosity values of gel containing *B.* *flabellifer* male flower ethanolic extract determined by a viscometer were 366.45 ± 20.01, 341.66 ± 28.91, and 326.2 ± 22.64 kcps, respectively. The viscosity of gel containing *B. flabellifer* male flower ethanolic extract, measured using a rheometer and a viscometer, decreased with the increasing extract concentration. This result might be due to the mild acidity of *B. flabellifer* male flower ethanolic extract (pH = 5.08 ± 0.3), which might reduce the neutralization capacity of triethanoloamine to carbopol.

**Figure 9:**
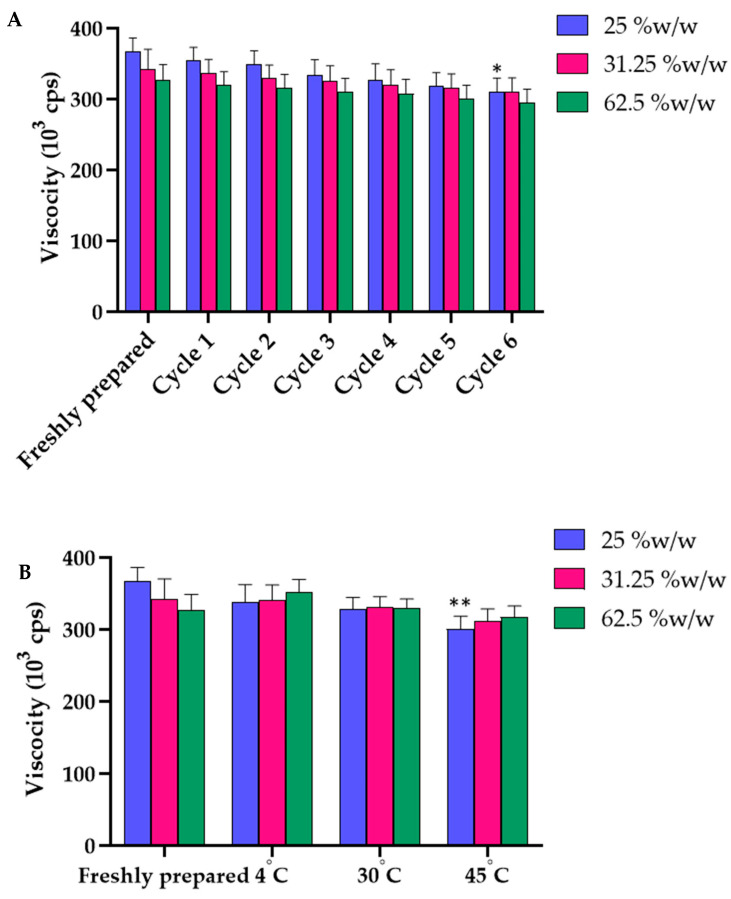
(**A**) Effects of six heating–cooling cycles on the viscosity of gel containing 25, 31.25 and 62.5% *w/w* of *B. flabellifer* male flower ethanolic extract. (**B**) Effect of temperature on the viscosity of gel containing 25, 31.25 and 62.5% *w/w* of *B. flabellifer* male flower ethanolic extract after storage at 4, 30 and 45 °C for 1 month. * Indicated *p* < 0.05 and ** indicated *p* < 0.01.

**Figure 10:**
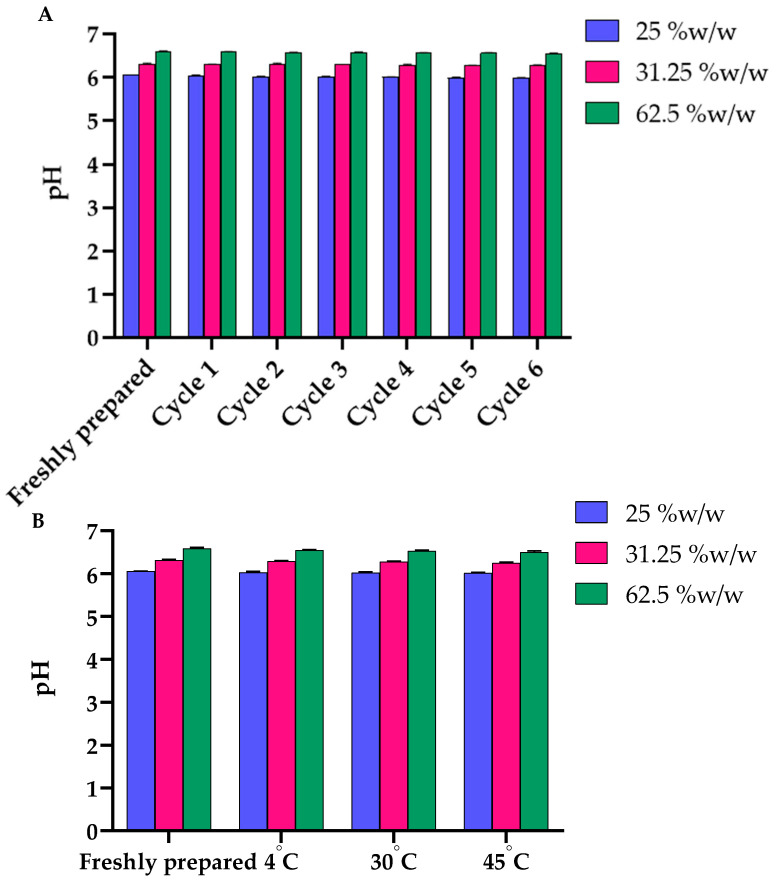
(**A**) Effects of six heating–cooling cycles on the pH of gel containing 25, 31.25 and 62.5% *w/w* of *B. flabellifer* male flower ethanolic extract. (**B**) Effect of temperature on the pH of gel containing 25, 31.25 and 62.5% *w/w* of *B. flabellifer* male flower ethanolic extract after storage at 4, 30 and 45 °C for 1 month.

The physical stability of all gel formulations containing *B. flabellifer* male flower ethanolic extract was confirmed by the maintained appearance, odor, color, and other physical characteristics. Gel separation and non-homogeneity were not observed in all formulations. After accelerated stability tests at all six cycles and 1-month storage at 4, 30, and 45 °C, the appearance, color, odor, and texture of the gel containing flower extract were not changed. The viscosity of the gel containing 31.25 and 62.5% *w/w* extract was not significantly changed, except the gel containing the lowest concentration of the extract (25% *w/w*) (Figure 9A). The viscosity of the gel formulation containing 25% *w/w* extract decreased significantly after the 6th heating–cooling cycle and after 1-month storage at 45 °C (Figure 9B). The pH values of all the formulations after they were freshly prepared and after the heating–cooling cycles and 1-month stability tests were not significantly different (Figure 10A,B).

## 3. Materials and Methods

### 3.1. Materials

Gallic acid, Griess reagent, DPPH (2,2-diphenyl-1-picrylhydrazyl), TPTZ (2,4,6-Tris(2-pyridyl)-s-triazine, ABTS (2,2′-azino-bis(3-ethylbenzothiazoline-6-sulfonic acid)), and coumarin were purchased from Sigma-Aldrich, St. Louis, USA. Absolute ethanol, dimethyl sulfoxide, sodium bicarbonate, sodium nitrate, and sodium hydroxide were purchased from RCI Labscan, Bangkok, Thailand. The Folin–Ciocalteu phenol reagent, aluminum chloride, sodium acetate trihydrate, ferrous sulfate heptahydrate (99% purity), and potassium persulfate were obtained from Loba Chemie, Mumbai, India. Quercetin (98% purity), epigallocatechin (EGCG) (98% purity), ascorbyl glucoside, phenoxyethanol, and triethanolamine were purchased from Chanjao Longevity Co., Ltd., Bangkok, Thailand. Clindamycin and diclofenac diethylammonium were purchased from RPC International Co., Ltd., Bangkok, Thailand. Carbopol Ultrez-21 polymer, propylene glycol, and glycerin were purchased from Namsiang, Bangkok, Thailand. Iron (III) chloride hexahydrate and 37% hydrochloric acid were purchased from Qrec, New Zealand.

### 3.2. Plant Material

**Figure 11:**
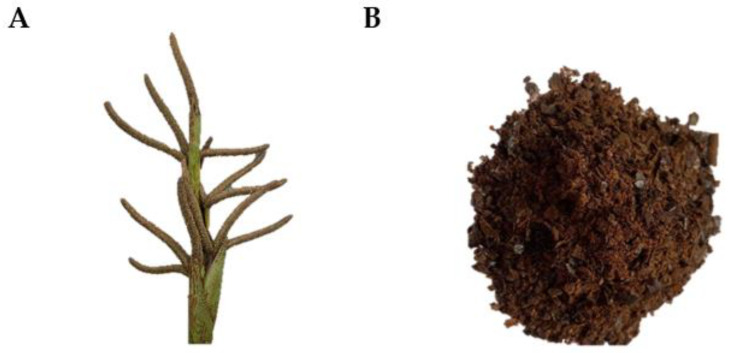
(**A**) Fresh male flower of *B. flabellifer.* (**B**) *B. flabellifer* male flowers dried extract.

The male flower of *B.* *flabellifer* was collected from Mueang District, Phetchaburi Province, Thailand. The 6-month-old *B. flabellifer* male flowers were harvested from a 5–20 year-old *B. flabellifer* tree at the end of April 2021 at 10:00–12:00 pm. The pictures of fresh and dried cut *B. flabellifer* male flowers were included in Figure 11. The sample has been identified by a botanist of the Forest Herbarium, Royal Forest Department, Bangkok, Thailand.

### 3.3. Preparation of Extraction of B. flabellifer Male Flower Ethanolic Extract

The male flowers of *B.* *flabellifer* were washed, cut into pieces, and dried at room temperature for 20 days. *B. flabellifer* male flowers were dried in a room with good ventilation to protect from direct exposure to light and heat from sunlight. The flowers were flipped several times during the day. The dried flowers were powdered and extracted by maceration of 300 g of powdered plant with 1700 mL of 90% ethanol at room temperature for 72 h. Then, the extract was filtered using filter paper and concentrated by evaporating the solvent using a rotary vacuum evaporator. The dried crude extract was weighed, and the yield was calculated by Equation (1).

(1) $$Yield\;\left(\%\right)=\frac{Weight\;of\;dried\;crude\;extract\;\left(g\right)}{Weight\;of\;dried\;plant}\times 100\%$$

### 3.4. Quantitative Analysis of Phenolic Compounds in the Ethanolic Extract of Male Flower of B. flabellifer by Folin–Ciocalteu Method

Total phenolic content was determined by the Folin–Ciocalteu reaction. Gallic acid solution at concentrations ranging from 7.8–125 µg/mL and *B.* *flabellifer* male flower extract solution at concentrations ranging from 7.8–250 µg/mL were added into 96-well plates (50 µL/well). Then, 10% *v/v* of Folin–Ciocalteu phenol reagent (100 µL) was added and mixed for 1 min. After 4 min of incubation, 7.5% *w/v* Na_2_CO_3_ solution (50 µL) was subsequently added to the mixture and incubated at room temperature in the dark for 2 h. Then, the absorbance was measured using a UV–visible spectrophotometer (Spectramax M3, Thermo Scientific, Waltham, MA, USA) at a wavelength of 765 nm. Total phenolic contents were calculated from the gallic acid standard curve. Data were expressed as µg gallic acid equivalents per mL of crude extract and µg/mg gallic acid equivalents of dry crude extract.

### 3.5. Quantitative Analysis of Total Flavonoid Content in B. flabellifer Male Flower Ethanolic Extract by the Aluminum Chloride Colorimetric Method

The total flavonoid contents in *B.* *flabellifer* male flower extract were determined by the aluminum chloride colorimetric method. Quercetin solution (3.9–1000 µg/mL), epigallocatechin solution (3.9–2000 µg/mL), and extract solution at concentrations of 3.9–500 µg/mL (100 µL/well) were added into 96-well plates. Then, 5% NaNO_2_ (30 µL) was added to the well and incubated for 5 min. Aluminum chloride (2% *w/v*, 50 µL) was added and incubated for 6 min followed by 10 min of incubation with 1 N NaOH (50 µL). The absorbance was measured at a wavelength of 510 nm with a UV–Vis spectrophotometer (Spectramax M3, Thermo Scientific, Waltham, MA, USA). Total flavonoid contents were calculated from quercetin and EGCG standard curves, and data were expressed as µg quercetin and EGCG equivalents per mL of crude extract and µg/mg quercetin and EGCG equivalents of dry crude extract.

### 3.6. Phytochemical Screening and Quantitative Determination of Bioactive Compounds in B. flabellifer Male Flower Ethanolic Extract by LC–MS/MS

The phytochemical screening of B. flabellifer male flower ethanolic extract was conducted using an LC–MS/MS instrument (Shimadzu Corporation, Kyoto, Japan) with ESI interface. The LC–MS/MS comprises a Column Oven: CTO-20A, Autosampler: SIL-20ACXR, Pump: LC-20ADXR, Degasser unit: DGU-20A3R, and Valve unit: FCV-20AH2. MS detection was performed using the LCMS 8040 model triple quadrupole mass spectrometer equipped with an ESI source operating in both positive and negative ionization modes and the data were processed by Lab solution software, version 5.82 SP1. The operation parameters were as follows: Heat block temperature of 400 °C, DL temperature of 250 °C, and autosampler temperature of 15 °C.

HPLC separation was performed on ACE Excel 5 Super C18 column (150 *×* 2.1 mm). Mobile phase consisted of two solvents: Solvent *(A)* deionized water with 0.1% formic acid and solvent (B) acetonitrile. Gradient elution was performed at a flow rate of 0.4 mL/min at 40 °C. A 5 μL injection volume of the sample was injected on to the column, separated, and eluted using the following gradients: 0.01 min (10% B); 2.00 min (50% B), and allowed for a column stabilization of 2 min with initial conditions.

### 3.7. Determination of DPPH Free Radical Scavenging Activity of B. flabellifer Male Flower Ethanolic Extract

The DPPH free radical scavenging capacity of the *B.* *flabellifer* male flower extract was determined and compared with gallic acid, quercetin, EGCG, and ascorbyl glucoside [44,45]. Gallic acid, EGCG, ascorbyl glucoside, and *B.* *flabellifer* male flower extract were diluted in deionized water, and quercetin was diluted in 95% ethanol at concentrations of 3.9–2000 µg/mL. DPPH was dissolved in absolute ethanol at a concentration of 500 µM. Gallic acid, quercetin, EGCG, ascorbyl glucoside, and *B.* *flabellifer* male flower extract (100 µL) were mixed with DPPH solution (100 µL). The mixture was incubated at room temperature in the dark for 30 min. The absorbance was measured using a UV–Vis spectrophotometer microplate reader at a maximum wavelength of 517 nm. The percentage of radical scavenging activity was calculated by Equation (2). The 50% of scavenging (IC_50_) was calculated from the non-linear regression analysis of the graph plotted between the percentages of DPPH free radical scavenging and the sample concentrations.

(2) $$DPPH\;Free\;radical\;scavenging\;\left(\%\right)=\frac{A-B}{A}\times 100\%$$

where *A* is the absorbance of the reaction with solvent control and *B* is the absorbance of the reaction with the extract.

### 3.8. Determination of ABTS Free Radical Scavenging Activity of B. flabellifer Male Flower Ethanolic Extract

Gallic acid, EGCG, ascorbyl glucoside, and *B.* *flabellifer* male flower extract were diluted in deionized water, and quercetin was diluted in 95% ethanol at concentrations of 3.9–2000 µg/mL. The ABTS scavenging assay was modified from a previous report [46]. ABTS was dissolved in absolute ethanol to a concentration of 7 mM. ABTS radical cation (ABTS^+^) was produced by the reacting ABTS stock. The solution with 2.45 mM potassium persulfate was dissolved in and remained in the dark at room temperature for 24 h prior to use. The ABTS^+^ solution was diluted with absolute ethanol to an absorbance of 0.700 (±0.02) at 734 nm. Gallic acid, quercetin, EGCG, ascorbyl glucoside, and *B.* *flabellifer* male flower extract (20 µL) were mixed with the ABTS solution (180 µL). The mixture was incubated at room temperature in the dark for 15 min. The absorbance was measured using a UV–Vis spectrophotometer microplate reader at a wavelength of 734 nm. The percentage of radical scavenging activity was calculated by Equation (3). The 50% of scavenging (IC_50_) was calculated from the non-linear regression analysis of the graph plotted between the percentages of ABTS^+^ free radical scavenging and the sample concentrations.

(3) $$ABTS\;Free\;radical\;scavenging\;\left(\%\right)=\frac{A-B}{A}\times 100\%$$

where *A* is the absorbance of the reaction with solvent control (deionized water) and *B* is the absorbance of the reaction with the extract.

### 3.9. Determination of Ferric Reducing Antioxidant Power of B. flabellifer Male Flower Ethanolic Extract

Gallic acid, EGCG, ascorbyl glucoside, and *B.* *flabellifer* male flower extract were diluted in deionized water, and quercetin was diluted in 95% ethanol at concentrations of 3.9–2000 µg/mL. The FRAP reagent was prepared from an acetate buffer (300 mM, pH 3.6), a solution of 10 mM TPTZ in 40 mM HCl, and 20 mM FeCl_3_ at 10:1:1 (*v/v*). The FRAP reagent (180 µL) and sample solutions (20 µL) were added to each well and mixed thoroughly. The mixture was incubated at 37 °C for 30 min. Then, the absorbance was read at 595 nm. The standard curve was constructed using a ferrous sulfate solution (9.8–5000 μM), and the results were expressed as μmol Fe (II) equivalent.

### 3.10. Antibacterial Activity of B. flabellifer Male Flower Ethanolic Extract against C. acnes

The study of the minimum inhibitory concentration (MIC) of *C.* *acnes* bacteria was performed by the broth microdilution method [47]. Samples were prepared by dissolving the extract in 95% ethanol at a concentration range of 0.244–500 mg/mL. The culture medium was added into 96-well plates, with 100 µL in each well. The prepared sample (100 µL) was added to the well and mixed with the culture. Clindamycin was used as a positive control at a concentration range of 0.024–50 µg/mL. *C.* *acnes* with turbidity 0.5 McFarland standard (100 µL) was added to the well. The culture was incubated at 37 ± 1 °C for 48 h in anaerobic conditions. The concentration of clindamycin and the extract that inhibited *C.* *acnes* growth completely (the first clear well) were taken as the MIC value.

### 3.11. Anti-Inflammatory Activity of B. flabellifer Male Flower Ethanolic Extract by the Albumin Denaturation Method

*B.flabellifer* male flower ethanolic extract was diluted with deionized water to obtain the concentrations of 0.25, 0.5, 1, 2, and 4 mg/mL. The extract solution (2 mL) was mixed with 0.2 mL of egg albumin (from fresh hen’s egg) and 2.8 mL of phosphate-buffered saline (PBS, pH 6.4). The reaction mixture was incubated at 37 ± 2 °C for 15 min and then heated for 5 min at 70 ± 2 °C. After cooling, the absorbance was measured at 660 nm using an UV–Vis spectrophotometer. Diclofenac sodium at the final concentration of (0.25, 0.5, 1, 2, and 4 mg/mL) was used as a positive control and treated similarly to determine absorbance. The percentage inhibition of protein denaturation was calculated using the following equation.

(4) $$Inhibition\;\left(\%\right)=\frac{V_{1}}{V_{0}-V_{1}}\times 100\%$$

where *V*_1_ is the absorbance of a test sample and *V*_0_ is the absorbance of control.

### 3.12. Formulation of Gel Containing B. flabellifer Male Flower Ethanolic Extract

The gel containing *B.* *flabellifer* male flower ethanolic extract was prepared by dispersing Carbopol Ultrez-21^®^ (0.7% *w/w*) in an aqueous medium containing propylene glycol (1.5% *w/w*) and glycerin (2.5% *w/w*). Then, the flower extract (25, 31.25, and 62.5% *w/w*) and phenoxyethanol (1% *w/w*) were added into the dispersion. Triethanolamine (0.8% *w/w*) was added dropwise until the gel was formed. The concentration of *B.* *flabellifer* male flower ethanolic extract used in this formulation was 1-, 1.25-, and 2.5-fold of MIC value against *C.* *acnes.* The extract was diluted with deionized water and filtered through a 0.45 µm syringe filter prior to use.

### 3.13. Characterization of Gel Containing B. flabellifer Male Flower Ethanolic Extract

The gel formulations containing *B.* *flabellifer* male flower ethanolic extract were inspected visually for appearance, color, odor, homogeneity, and consistency. The gel’s viscosity and pH were determined using a viscometer (Yanhe, China) and pH meter (Eutech Instruments, Singapore), respectively.

### 3.14. Rheological Measurement

The measurement of gel containing *B.* *flabellifer* male flower ethanolic extract rheology was performed using a Thermo Scientific HAAKE RheoStress 1 rheometer equipped with a plate and plate geometry (1.0 mm gap, 60 mm diameter). The temperature of the gel was set at 25 °C. The range of shear rate was from 0.01 to 200 s^−1^ with the frequency of 1 Hz.

### 3.15. Accelerated Stability of Gel Containing B. flabellifer Male Flower Ethanolic Extract

The accelerated stability study of gel formulations containing the flower extract was performed by heating–cooling cycles. The test was performed in 12 days with six cycles. In each cycle, the freshly prepared gel containing extract was kept at 4 °C for 24 h and 45 °C for another 24 h [48]. The pH and viscosity of the gel after each cycle were measured every cycle. In addition, the stability of all gel formulations was determined by maintaining the products at 4, 30, and 45 °C for 1 month. Then, the pH and viscosity of gels were measured.

### 3.16. Statistical Analysis

Data were presented as mean ± standard deviation (SD). The one-way analysis of variance (ANOVA) and Tukey post hoc test were performed for statistical analysis. To compare the significance of the difference between the means of two groups, a *t*-test was performed. A *p*-value < 0.05 was considered statistically significant.

## 4. Conclusions

The *B.* *flabellifer* L. male mature flower extract contained high concentrations of total phenolic and flavonoid contents. The bioactive compounds found in the extract were gallic acid, coumarin, and quercetin. The phytoconstituent of *B.* *flabellifer* L. male flower extract was shown to be correlated with the antioxidant activity of the extract. The *B.* *flabellifer* L. male flower extract had antibacterial activity against *C.* *acnes* with the MIC value of 250 mg/mL. At 250 and 500 µg/mL, the extract demonstrated the anti-inflammatory activity by preventing albumin denaturation. The anti-acne gel containing the *B.* *flabellifer* male flower ethanolic extract was initially and successfully developed with acceptable physicochemical properties and stability.

## Figures and Tables

**Table 1:** Identification and quantification of compounds of ethanol extract of *B. flabellifer* male flower by LC–MS/MS.

Compound	Precursor Ions (*m/z*)	Product Ions		Retention Time (min)	Equation	R^2^	Linearity (µg/mL)	Quantification of *B. flabillifer* Male Flower (µg/mL)
		Target Ion	Reference Ion					
Gallic acid	169.05	125.05	79.05	1.69	Y = (762,191)x + (50,587.9)	R^2^ = 0.9989	0.250–5.000	1.610
EGCG	459.10	139.10	289.05	3.68	Y = (605,393)x + (−1614.28)	R^2^ = 0.9980	0.005–0.200	Not detected
Quercetin	303.05	153.15	229.10	4.16	Y = (508,792)x + (24,227.3)	R^2^ = 0.9992	0.250–5.000	0.912
Coumarin	147.05	91.15	103.10	4.62	Y = (593,159)x + (−37.6301)	R^2^ = 0.9999	0.005–0.200	0.021

**Table 2:** Pearson correlation coefficients of total phenolic and flavonoid contents and antioxidant activities of *B.* *flabellifer* male flower ethanolic extract measured by DPPH, ABTS, and FRAP assays.

	Total Phenolic Content (Gallic Acid Equivalent)	Total Flavonoid Content (Quercetin Equivalent)	Total Flavonoid Content (EGCG Equivalent)
DPPH assay	0.8256 *	0.7001	0.6962
ABTS assay	0.9964 ***	0.9932 ***	0.9932 ***
FRAP assay	0.9972 ****	0.9970 ****	0.9965 ****

* Indicated *p* < 0.05, *** indicated *p* < 0.001, and **** indicated *p* < 0.0001.

**Table 3:** Appearance of *B. flabellifer* male flower ethanolic extract and gel containing 25, 31.25, and 62.5 %*w/w B.* *flabellifer* male flower extract.

Concentration of *B. flabellifer* Male Flower Ethanolic Extract	25% *w/w*	31.25% *w/w*	62.5% *w/w*
Extract (dissolved in 50% ethanol)	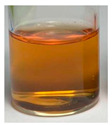	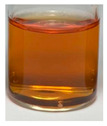	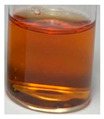
Gel containing extract